# Being barefoot. Prevalence at home, in school and during sport: a cross-sectional survey of 714 New Zealand secondary school boys

**DOI:** 10.1186/s13047-018-0285-y

**Published:** 2018-07-18

**Authors:** Peter Francis, Grant Schofield, Lisa Mackay

**Affiliations:** 1 Musculoskeletal Health Research Group, School of Clinical and Applied Sciences, Leeds Beckett University, Leeds, UK; 2 Human Potential Centre, School of Sport and Recreation, Auckland University of Technology, Auckland, New Zealand

**Keywords:** Barefoot, Shoes, Footwear, Running, Athletics

## Abstract

**Background:**

It may be assumed that a combination of culture, climate and economic resource are the major reasons that non-industrialised countries have a higher prevalence of barefoot activity. New Zealand is an industrialised country with comparable resources to that of many European countries; however, it seems to remain socially acceptable to carry out barefoot activities. A chance observation of students competing barefoot on a tartan track, prompted us to determine the prevalence of barefoot activity in an all-boys secondary school in Auckland New Zealand.

**Method:**

An 11-question survey was administered at an Auckland boys secondary school, of high socioeconomic status, to determine the footwear habits of students (*n* = 714) during: a) daily life b) school life (c) physical education class and (d) sport. To classify students as habitually barefoot or shod, students were asked to select whether they were barefoot most of the time (2-points), half of the time (1-point) or none of the time (0-points) in three settings: around the house, during sport and during school. A score of ≥3 was required to be considered habitually barefoot. Participants were also asked to specify, when running at their most recent athletics event (100 m – 3000 m) on a track, whether they ran barefoot, in shoes, in spikes or another type of footwear. Finally, participants were asked to indicate if leg pain had interrupted running during the previous 12-months. Analysis was conducted using IBM SPSS.

**Results:**

45% (95% CI: 41.5–49.5%) of the participants in our sample were classified as habitually barefoot. More than half of the sample reported being barefoot most of the time at home (*n* = 404, 56.6%) and during PE class (*n* = 420, 58.8%). Over 50% of the sample reported being barefoot half of the time or more during sport (*n* = 380, 53.2%). A smaller amount went to the supermarket (*n* = 140, 19.6%) or took the bus (*n* = 59, 8.3%) whilst barefoot around half of the time or more. The percentage of barefoot competitors declined with increasing distance: 100 m (46.5%), 200 m (41.8%), 400 m (38%), 800 m (31%), 1500 m (31%) and 3000 m (20%). The prevalence of leg pain interfering with running was 23.5%. There was no difference in the prevalence of leg pain between those classified as habitually barefoot and shod (Χ^2^(1, *N* = 603) = 0.005, *p* = 0.946).

**Conclusion:**

The results of this survey demonstrate that over 50% of students at an all-boys secondary school in Auckland, of high socioeconomic status, are barefoot at home, during physical education and sport half of the time or more. These results may point towards a cultural difference between New Zealand and other modern industrialised countries.

## Background

Humans were barefoot for millions of years during evolutionary history. During the past few centuries, particularly in industrialised countries, walking and running have generally been carried out in shoes. [1] Recent evidence [2] supports empirical data suggesting that differences in foot morphology occur between those who grow up habitually barefoot or shod. [3–7] The main differences being a reduction in arch height and a narrower foot in those who grow up shod. Increasing time spent barefoot is thought to reverse some of these changes. [8] Comparisons in foot morphology are normally made between shod populations from industrialised countries and barefoot populations from non-industrialised countries where climate, culture and lower economic resource are assumed to be the primary reasons for the absence of footwear. [2] At present, the consequences of developmental differences in foot morphology, because of growing up habitually barefoot or shod, on motor learning or longer-term health are unknown.

In recent years, the scientific debate in relation to the presence or absence of footwear has focused on differences in gait and injury incidence between shod and barefoot runners. [9, 10] The kinematic changes reported when transitioning from shod to barefoot running are thought to be driven by a highly sensitised plantar surface of the foot particularly at the heel and metatarsal-phalangeal joints. [11] Stimulation of the foot in these regions results in plantar and digit flexion, which in a load bearing position is designed to distribute load toward the lateral edge of the foot and distal digits avoiding painful sensations at the heel. [8] At present, there has not been sufficient longitudinal investigations to determine whether differences in gait kinematics arising from altered foot sensation leads to differences in injury incidence between shod and barefoot runners.

**Fig. 1:**
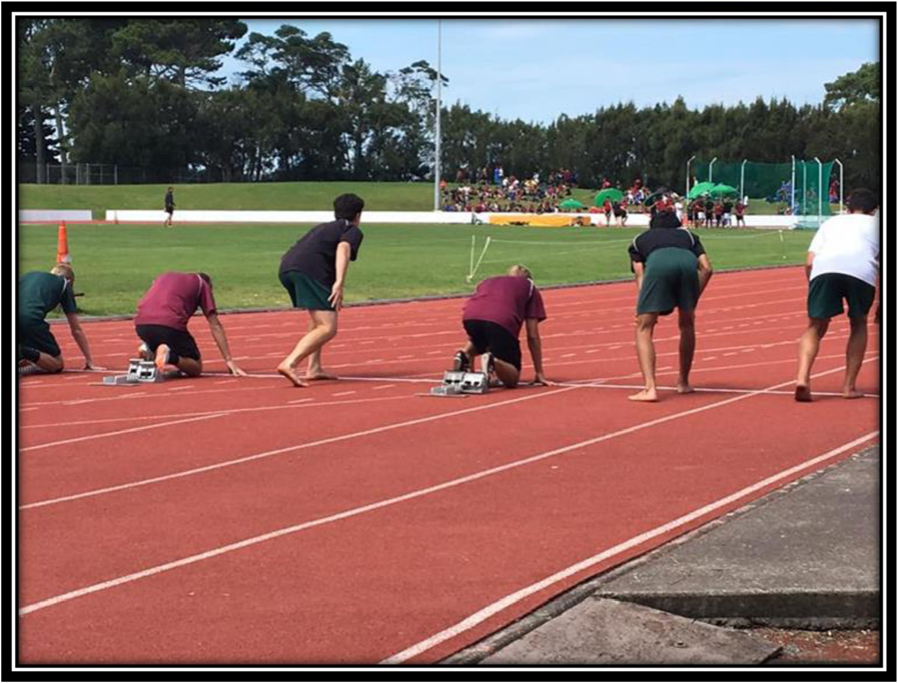
The start of the 100 m in a secondary school boy’s athletics event in Auckland, New Zealand, March 2017

Footwear, particularly that used in repetitive athletic activities, represents an economic cost to communities and is often purchased in the interest of maintaining musculoskeletal health or injury prevention. However, in running at least, the evidence to support the prescription of specific shoe types, with the aim of injury prevention, is inconclusive. [12, 13] Given that the health implications of differences in foot morphology and running kinematics because of growing up shod are unknown, it raises the question should children and adolescents be advised to always wear shoes in circumstances where there is little threat to foot perforation or extreme temperatures? New Zealand is an industrialised country with comparative resources to American and European countries used to study shod populations. However, it appears to be culturally apparent and socially acceptable to conduct activities barefoot. The authors of this paper observed secondary school students (Fig. 1), from an area of high-socioeconomic status, competing barefoot on what is known to be a hard and roughened surface (tartan track) during an athletics meet. These observations prompted us to question the footwear practices of New Zealand adolescents taking part in athletic activities. The primary aim of this study was to determine the prevalence of barefoot activity; during a) daily life b) school life (c) physical education class (d) sport and (e) when running in their most recent schools athletics event; in a sample of boys attending a secondary school in Auckland New Zealand. A secondary aim was to estimate the prevalence of lower limb pain, which had interrupted running-related activities in the previous 12-months.

## Method

### Study design

This study was a cross-sectional survey targeting an all-boys secondary school in Auckland. New Zealand. The Auckland University of Technology research ethics committee approved this study (AUTEC Reference number 17/222).

### Participants and setting

Eligible participants were all boys (12–19 years) attending Westlake Boys’ High School in Auckland, New Zealand. Information on the size and socio-economic status of the school was obtained from the 2017 Ministry of Education school roll returns. The survey was advertised to the teacher with the responsibility of ‘Head of Sport’, via a follow up email following the school athletics day, which took place at the Auckland University of Technology athletics track. A meeting was arranged to deliver 1000 paper copy surveys to the school. Completed surveys were collected from the school 1-week later.

### Measurements

All participant data was collected via a custom developed, paper-based, 11-question survey (Appendix 1). The survey gathered demographic (school year and ethnicity), footwear, competition and pain related data. Participants were asked to indicate the time they spent barefoot during school, during PE class, during sport, around the house, at the supermarket and on the bus. Time spent barefoot was classified as most of the time, half of the time, or none of the time to determine the number of students ‘habitually barefoot’ and allow comparison with the work of Holllander et al. [2] in Germany and South Africa. Using three items (school, sport, and around the house), participants were given 2-points for being barefoot most of the time, 1-point for being barefoot half of the time and 0-points for being barefoot none of the time. A minimum of 3-points was required from a maximum of 6 to be classified as habitually barefoot. Participants were asked to report the surface of their most recent athletics event and whether they competed barefoot, in shoes, spikes or other. Subsequently, participants were asked to specify the event (s) they competed in at their most recent athletics event on a track. Participants were then asked to specify for the events between 100 m and 3000 m whether they competed barefoot, in shoes, spikes or other. Finally, participants were asked whether they competed in an athletics club outside of school and whether leg pain or an injury had stopped them running during the last 12 months.

### Procedure

The surveys were delivered and explained to a teacher (Head of Sport) on a Friday (23rd June, 2017) and collected 7-days later (30th June, 2017). This teacher distributed the survey to all other teachers to administer in class. This was to ensure the maximum number of boys were recruited and as such, that the sample obtained was representative of the observations made at the track (Fig. 1). Teachers were provided with study information to explain to the students so that they could provide informed consent. Students who wished to participate, completed the survey and returned it to their teacher.

### Analysis

Survey responses were manually entered in Qualtrics Survey Software. Data were then imported into SPSS for analysis (IBM SPSS Statistics for Windows, Version 24. Armonk, NY: IBM Corp.) for analysis. The prevalence of barefoot activity was calculated as the proportion of those classified as habitually barefoot from valid survey responses. The 95% Confidence Interval (CI) was calculated based on the standard Wald method. Prevalence estimates were also calculated for the total study population for barefoot settings, athletics participation (event, surface, and footwear), and occurrence of leg pain. Chi-square tests of independence were performed to examine the relation between being habitually barefoot and ethnicity, and with leg pain.

## Results

The school was the second largest boys’ school (*n* = 2312) and sixth largest school overall from 90 secondary schools in the Auckland region. The school was part of a community of high socio-economic status (Decile 9/10). A total of 714 students responded to the survey. Table 1 displays a breakdown of participants’ school year and ethnicity considering missing data from non-completion of some questions or early exit from the survey.

**Table 1:** Participant school year and ethnicity, n (%)

	Total Responses *n* = 714
School Year	
9	178 (25)
10	229 (32)
11	123 (17)
12	110 (15)
13	46 (6)
N/A	28 (4)
Ethnicity	
Māori	59 (8)
Pacific Peoples	50 (7)
Asian	193 (27)
^a^MELAA	41 (6)
^b^NZ European/Other	340 (48)
N/A	31 (4)

^a^Middle Eastern, Latin, American, African

^b^New Zealand European

**Fig. 2:**
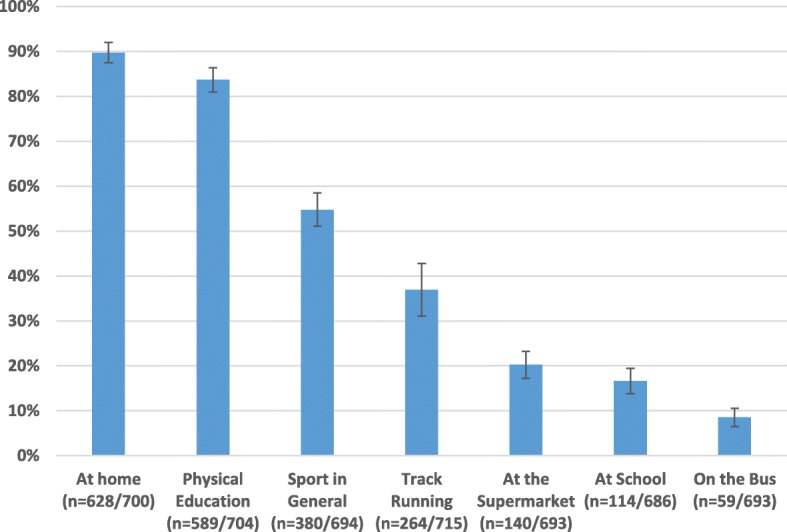
The proportion (95% CI) of secondary school boys who were barefoot half of the time or more

Forty-five percent (95%CI: 41.5–49.5%) of the sample were classified as habitually barefoot. There was a small difference in the prevalence of habitual barefoot activity between ethnicities: Māori (*n* = 12/23, 52%), Pacific Peoples (*n* = 17/37, 46%), Asian (*n* = 62/170, 36%), MELAA (n = 12/38, 32%), and NZ European/Other (*n* = 189/380, 50%), Χ^2^(5, *N* = 678) = 11.968, *p* = .035, Cramer’s V = .133. Table 2 displays the prevalence of barefoot frequency at home and at school; during physical education class and sport; and whilst at the supermarket or on the bus. Over 50% of the sample were barefoot at home, during physical education and sport for at least half of the time or more (Fig. 2).

**Table 2:** Prevalence of barefoot frequency among 714 secondary school boys n (%)

	Home	School	Physical Education	Sport	Supermarket	Bus
Most of the Time	404 (57)	26 (4)	420 (59)	98 (14)	38 (5)	32 (4)
Half of the Time	224 (31)	88 (12)	169 (24)	282 (39)	102 (14)	27 (4)
None of the Time	72 (10)	572 (80)	115 (16)	314 (44)	553 (77)	634 (89)
N/A	14 (2)	28 (4)	10 (1)	20 (3)	21 (3)	21 (3)

Of the students surveyed, 91% (*n* = 639) had competed in a schools’ athletics event and 11% (*n* = 75) competed in athletics outside of school. The venues that students had competed at in their most recent athletics events included a grass track (*n* = 172, 24%), a non-grass track (*n* = 190, 27%), a field (*n* = 175, 25%), a road (*n* = 20, 3%) and others (*n* = 92, 13%). Students competed in shoes (*n* = 394, 55%), spikes (*n* = 50, 7%), barefoot (*n* = 264, 37%) and others (*n* = 11, 1.5%). On a track (Fig. 1), during their most recent athletics event, students ran in the 100 m (*n* = 430, 60%), 200 m (*n* = 282, 40%), 400 m (n = 172, 24%), 800 m (*n* = 129, 18%), 1500 m (*n* = 145, 20%) and 3000 m (*n* = 103, 14%).

**Fig. 3:**
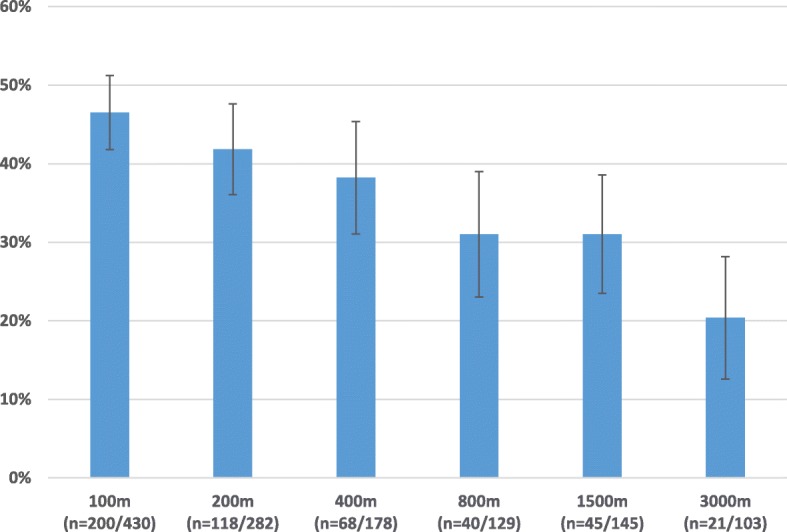
The proportion of barefoot competitors (100–3000 m) on a tartan track

More than 40% of students competing in the 100 m (*n* = 200/430) and 200 m (*n* = 118/282) competed barefoot. Over 30% of boys ran in the 400 m (*n* = 68/172), 800 m (*n* = 40/129) and 1500 m (*n* = 45/145) barefoot. The number of barefoot competitors decreased with increasing distance (Fig. 3) such that ~ 20% (*n* = 21/103) of the 3000 m field competed barefoot.

The prevalence of leg pain that had stopped students running during the past 12-months was 23.5% (*n* = 168). A Chi-square test for independence indicated no significant association between being habitually barefoot and leg pain, Χ^2^(1, *N* = 603) = 0.005, *p* = .946.

## Discussion

The aim of this study was to estimate the habitual time spent barefoot by boys attending a secondary school in Auckland, New Zealand. The school was one of high socioeconomic status and ethnically representative of the Auckland region as reported in the most recent census. [14] Based on the definition of Hollander et al. [2], 45.3%of boys were classified as habitually barefoot. The prevalence of habitual barefoot activity in our New Zealand sample is between that of almost completely (90.9%) barefoot South Africa and completely (100%) shod Germany. The German and South African data comes from school aged (primary and secondary) children and adolescents (*n* = 849) surveyed by Hollander et al. [2]. Cultural and climatic differences are likely to contribute to differences in footwear habits between German and South African children. However, South Africa’s lower world ranking in terms of economic resources compared to Germany (93rd vs. 19th) will also contribute to most children in South Africa being habitually barefoot. [15] By contrast, New Zealand is an industrialised country ranked closer to Germany (35th vs. 19th) in terms of economic resources. Despite similar economic wealth, 50% of boys in our sample from an area of high socioeconomic status were barefoot at least half of the time at home, during physical education and sport. This may suggest that cultural and climatic differences are the primary reasons for differences in footwear habits between German and New Zealand students. New Zealand and Germany both share a temperate climate in contrast to South Africa’s mixed arid and temperate climate. [16] It is plausible that a greater mean temperature in New Zealand encourages more barefoot activity. However, if that were solely the case, similar behaviour would be prevalent in all industrialised countries with a warmer climate. To the best of the author’s knowledge and in the absence of global data, barefoot activity is not prevalent amongst all students in warmer industrialised countries. This could suggest cultural differences in New Zealand as a primary factor in the differences we report. This suggestion is supported, albeit weakly, by the within sample cultural difference between ethnic groups. The ethnic groups strongly associated with New Zealand (Māori, Pacific Peoples, New Zealand European) spent a greater proportion of time barefoot compared to the other (Asian, MELAA) ethnicities (46–52% vs. 32–36%).

Many of the students (*n* = 404) spent most of their time at home barefoot. This might be expected to be similar in many countries regardless of climate due to the more predictable surface and warmth associated with the indoors. However, just as many of the students (*n* = 420) participated in physical education class barefoot, again pointing towards a cultural difference. This is added to by the fact that over half of the sample participate in all sports barefoot for at least half of the time. The fact that the majority of students (77–89%) are shod attending school classes, the supermarket and getting on a bus may point towards school policy and different levels of social acceptability depending on the context of the environment. It is interesting to note that 140 students attended the supermarket half of the time or more in bare feet. It remains to be seen whether the prevalence of this behaviour (~ 19%) would be replicated in other industrialised countries of warmer climate.

This study was conceptualised due to the observation of students competing barefoot on a tartan track. We are confident we sampled our intended participants due to 91% having competed in a school athletics events. The head teacher being aware of the students who participated in the event we had observed will have assisted us in identifying the target sample. Almost a third and up to half of students were willing to compete in events between 100 m (47%) and 1500 m (31%) barefoot. Although the number of students competing barefoot declined with increasing distance, 20% of the field in the 3000 m were willing to compete barefoot (*n* = 21/103). Our findings suggest that secondary school boys in Auckland New Zealand spend a considerable amount of time barefoot during activities of daily living and are willing to run, at least for short distances, on hardened surfaces.

The prevalence of leg pain or injury that interfered with running in the previous 12-months was lower than that reported by high school cross-country athletes (48% vs. 24%) over the course of one season in the United States. [17] However, this comparison may not be appropriate given that although over 90% of our sample had competed in a schools athletics event, only 11% regard themselves as athletes (athletics) outside of school. Compared to the overall prevalence of injury (34–38%) sustained from physical activity in school aged (middle and high) students in the United States our findings in relation to leg pain may be considered on the lower end of the spectrum. [18] Furthermore, we consider the prevalence of pain to be low in our sample as it is drawn from a school with a winter and summer sports programme including multiple team sports that are fielded at participation and elite level. There was no difference in exercise related leg pain, which interfered with running between those habitually barefoot compared to those habitually shod. In the absence of sport participation and or training / competition exposure data, we cannot make inferences between footwear habits and pain or injury from our data set. However, our findings suggest that adolescents who spend considerable amounts of time barefoot and are willing to compete on hardened surfaces while barefoot, do not seem to report a higher prevalence of pain or injury than reported from youth sport generally.

Our study is the first to report the footwear habits in boys attending a secondary school in New Zealand. However, despite sampling from the intended population, our sample still only represents ~ 31% of those attending the school. As a result, we may have missed some of the competitors witnessed on the track in Fig. 1. However, the declining numbers with increasing year group may point toward lower sports participation among the older students as much as it does missing data. Our sample comes from one secondary school in Auckland and despite similar ethnic representation when compared to census data, our findings cannot be considered generalizable to all boys in Auckland or New Zealand as a whole. Differences in temperature between the north and south-island in addition to Auckland being the country’s largest urban area could affect footwear habits elsewhere. The questions in our survey rely on the participants considering barefoot as without socks, it is possible for the question in relation to being barefoot around the house, the boys might have considered being in socks as the same thing. Linked to this point, participants who report being barefoot at home automatically score 2-points and therefore, are only required to be barefoot half of time at school or during sport to be considered habitually barefoot. Future work may need to consider whether this is the most valid method of classification. We used this method to allow comparison with German and South Africa samples [2] and we would suggest that it is reasonable to regard being barefoot half of the time during school or sport as ‘unusual’ for industrialised countries, an assertion that is supported by the German data. The prevalence of leg pain interfering with running in the previous 12-months must be interpreted cognisant of the fact that we did not collect sport participation or exposure data. It is possible that the low prevalence is due to low participation outside of schools athletics events.

## Conclusion

New Zealand is an industrialised country with similar economic resources to some of the world’s richest nations. Despite this, ~ 45% of boys attending a secondary school in a community of high socio-economic status were habitually barefoot. This contrasts with other industrialised nations such as Germany, where previously, an absence of barefoot activity has been reported. In the school’s athletics event, 31 to 47% of competitors ran barefoot on a hard surface for distances up to 1500 m. The prevalence of exercise related leg pain was no higher than that reported previously in youth sport. The findings of this study question whether the prescription of athletic shoes, given their economic cost, is necessary in situations where risk of perforation to the skin and extreme temperatures are absent. Although this study design was specific to the target sample, future studies should seek to add to global data on footwear habits in adolescents, at least in relation to a) daily life b) school life (c) physical education class and (d) sport. Longitudinal investigations on the impact of footwear habits on foot morphology and musculoskeletal health are required.

## Abbreviations

CI: confidence interval
MELAA: Middle Eastern, Latin American or Africa
NZ European: New Zealand European
